# Key Nutritional Considerations for Youth Winter Sports Athletes to Optimize Growth, Maturation and Sporting Development

**DOI:** 10.3389/fspor.2021.599118

**Published:** 2021-01-27

**Authors:** Marcus P. Hannon, Joelle Leonie Flueck, Vincent Gremeaux, Nicolas Place, Bengt Kayser, Chris Donnelly

**Affiliations:** ^1^Research Institute for Sport and Exercise Sciences (RISES), Liverpool John Moores University, Liverpool, United Kingdom; ^2^Swiss Paraplegic Centre, Institute of Sports Medicine, Nottwil, Switzerland; ^3^Swiss Olympic Medical Center, Centre Hospitalier Universitaire Vaudois, Lausanne, Switzerland; ^4^Institute of Sport Sciences, University of Lausanne, Lausanne, Switzerland

**Keywords:** Winter Youth Olympic Games, energy, macronutrients, micronutrients, iron, calcium, vitamin D, youth athlete

## Abstract

Despite a wealth of sport nutrition guidelines for adult athletes, there are currently no nutrition guidelines for youth winter sports athletes. Whilst it may be pragmatic to apply nutrition guidelines for adult athletes to youth winter sports athletes, it is inappropriate. Due to a paucity of research on youth athletes, it is impossible to provide evidence-based guidelines for this population, so careful extrapolation from the theoretical and practical considerations that apply to other athletic groups is necessary. Youth winter sport athletes undergo rapid biological growth and maturation which influences their nutritional requirements. A varied and balanced diet that ensures sufficient energy availability for optimal growth and maturation as well as sporting performance is the cornerstone of youth athlete nutrition and should also allow for youth athletes to meet their micronutrient requirements. In some cases, micronutrient status (e.g., vitamin D and iron) should be monitored and optimized if appropriate by a medical professional. Dietary supplement use is prevalent amongst youth athletes, however is often unnecessary. Education of youth athletes, their parents and coaches on best nutritional practices as well as the risks associated with dietary supplements is vital for their long-term athletic development. Further research in youth winter sports athletes across different stages of growth and maturation competing in a variety of sports is urgently required in order to inform nutritional guidelines for this population.

## Introduction

Despite a wealth of sport nutrition guidelines for adult athletes (e.g., Thomas et al., 2016), there are only a few review papers for winter sports athletes (e.g., Meyer et al., 2011) and even fewer original research papers on the nutrition needs or practices of youth winter sports athletes. Whilst it may be pragmatic to simply apply recommendations for adult athlete's to youth athletes, there are many reasons why this is inappropriate (see Hannon et al., 2020b for an overview). As a result of ongoing growth and maturation, youth athletes go through many anatomical and physiological changes that impose specific nutritional requirements during their second decade of life (Malina et al., 2004). Nutritional recommendations for youth athletes therefore should not only focus on sporting performance but first meet the requirements for optimal growth and development (Bergeron et al., 2015, Tercier et al., 2019).

Youth winter sports athletes have diverse physiological and metabolic capacities, their respective sporting demands, physiological and metabolic, differ greatly and they compete in a wide variety of conditions. The unique combination of maturity status (Lloyd et al., 2014), sport [e.g., ice hockey (Konarski et al., 2019), ski mountaineering (Praz et al., 2014) or curling (Ainsworth et al., 2000)] and environment [e.g., indoor or outdoor, often cold but sometimes hot, at low or high altitude (Ocobock, 2016)] poses key challenges for practitioners working with youth winter sports athletes. This narrative mini-review discusses some of these key considerations and challenges using the (limited) available literature, and provides some practical approaches with which they can be addressed (Table 1). The extant literature was searched using Scholar, PubMed, Web-of-Science, and Scopus from inception to December 2020 using database adapted search strings based on the key-words nutrition, energy expenditure, athletes, youth, winter, sport, and combinations thereof.

**Table 1:** Key messages, practical considerations and important education messages on energy, vitamin D, iron and hydration for the youth winter sport athlete.

**Energy**	**Importance for the youth winter sports athlete:**
	• Sufficient energy availability is essential for optimal growth, maturation, and sporting performance (>45 kcal^−1^ · kg FFM^−1^·day^−1^ for adults).
	**Practical considerations and application:**
	• The energy requirements of youth winter sport athletes vary considerably. Consider each athlete's anthropometric profile, rate of growth, NEAT, and sporting demands (including training and competition load).
	• Monitor rate of growth (stature and body mass) and maturation (maturity offset, i.e., time from PHV)-3–4 times per year.
	• Be alert to any symptoms of low energy availability (RED-S) such as chronic fatigue sensation, low mood, reduced performance, poor concentration, impaired immune system and in females, absence of menarche.
	**Education:**
	• Energy density and macro-/micronutrient content of different foods.
	• Weighing out and visualizing a “typical day's food.”
	• Importance of not missing meals and snacks.
	• Planning nutrition into their schedule (e.g., when traveling or at school).
**Vitamin D**	**Importance for the youth winter sports athlete:**
	• Winter sports athletes are at risk of vitamin D deficiency. This prohormone, alongside calcium, is required in sufficient quantities for bone mineral accrual to ensure optimal skeletal growth and development.
	Practical considerations and application:
	• Assess vitamin D status and supplement vitamin D_3_ accordingly. Vitamin D status can be obtained via a simple finger prick blood sample (and appropriate analysis), or regular blood lab analysis.
	• If unable to determine vitamin D status a safe dose (typically 800 IU per day−5600 IU per week) may be beneficial to prevent deficiency during winter months.
	• Consider ethnicity, habitual latitude, and frequency of skin exposure to sunlight.
	**Education:**
	• Difficult to be obtained in sufficient amounts through diet.
	• Importance of (safe) sunlight exposure.
	• Need for correcting a deficiency through safe sunlight exposure and/or supplementation.
**Iron**	**Importance for the youth winter sports athlete:**
	• Iron is an important mineral for many biological processes. Iron requirements increase as a result of (tissue) growth and in females increase further due to menarche.
	**Practical considerations and application:**
	• High prevalence of iron deficiency in youth athletes due to insufficient dietary iron intake.
	• Iron requirements may be increased at altitude, which is relevant for altitude training camps.
	• Non-haem iron (primarily from non-animal sources) has a low bioavailability making vegetarian/vegan athletes at greater risk of iron deficiency.
	• Iron absorption is enhanced when consumed with vitamin C and is impaired when consumed alongside tea and coffee.
	• In case of deficiency iron supplementation should be prescribed by a qualified clinical practitioner.
	**Education:**
	• Iron rich foods and their bioavailability.
	• Food items and combinations that inhibit or favor iron uptake.
	• Symptoms of iron deficiency (e.g., unexplained fatigue sensation, loss of concentration, diminished performance).
**Hydration**	**Importance for the youth winter sports athlete:**
	• There are differences in thermoregulation mechanisms between adult and youth athletes, and among adolescents depending on maturation state. Furthermore, differences in environment, clothing, and metabolic demands between sports lead to differences in heat dissipation and subsequent fluid requirements.
	**Practical considerations and application:**
	• Consider environment (i.e., temperature, humidity, and altitude) in addition to clothing.
	• Appropriate fluid availability (whilst considering environmental temperature) during training and competition, ensuring enough fluids are consumed to prevent excessive dehydration (i.e., >2% of body mass).
	• Add flavorings and use hot or cold drinks when appropriate to increase palatability and consumption.
	• Check urine color (aim for pale colored urine).
	• Check body weight pre- and post-training session/competition to assess fluid losses and determine optimal drinking strategy (including rehydration at 1.5 L fluids per kg body mass lost).
	**Education:**
	• Urine color charts to illustrate hydration/dehydration.
	• Individualized fluid loss assessment.
	• Optimal drinking scheme for the specific sport.

*NEAT, non-exercise activity thermogenesis; RED-S, relative energy deficiency in sport; PHV, peak height velocity. Please see main text for references*.

### Key Physiological Changes in Youth Winter Sports Athletes That Influence Their Nutritional Needs

Youth winter sports athletes (15–18 years) cannot simply be considered “mini adult athletes” despite sometimes competing against adults. Except for the first year of life, adolescence (typically 10–19 years) is the period of greatest growth, maturation, and development across the lifespan (Malina et al., 2004). Growth describes an increase in size whereas maturation describes a more global aspect of physical and cognitive development. More specifically, maturation is the progress toward a biologically mature state, and the rate at which it proceeds is highly variable between different organ systems and tissues (Malina et al., 2004). For example, sexual maturation (the progress toward fully functional reproductive capability) differs from skeletal maturation (the progress toward the skeleton becoming fully ossified) and somatic maturation (the progress toward adult stature; Malina et al., 2004).

There is also a large inter-individual variation in biological age, which may vary (by up to 4 years) from chronological age–the time interval since birth (Lloyd et al., 2014, Malina et al., 2015). Key phases of growth and physical development (i.e., pre-, circa- and post-peak height velocity) lead to many changes such as increases in body mass, muscle mass and blood volume and accrual of bone mass and density all of which influence nutritional requirements (Unnithan and Goulopoulou, 2004). Anthropometric differences between male and female adolescents are the main driver of sex differences in the nutritional requirements of this population, with one exception being iron (see Iron section; Unnithan and Goulopoulou, 2004).

Several major factors must therefore be considered when working with youth athletes: (1) their current maturity status and rate of growth and maturation, (2) their current physiological, metabolic, and psychosocial capacities, and (3) their general life and sport demands (Bergeron et al., 2015, Hannon et al., 2020b). Understanding these factors and their inter-play is a crucial step to developing sport-specific nutritional guidelines (i.e., recommended macro- and micro-nutrient intake) for youth athletes (Bergeron et al., 2015). For example, growth and maturation increase the size of glycogen stores and the relative energy contribution from anaerobic metabolism during exercise, but decreases relative rates of exogenous carbohydrate substrate utilization during exercise (for reviews see Armstrong et al., 2015, Ratel and Blazevich, 2017). These factors may lead to an increased reliance on carbohydrate for energy supply in early compared with late adolescence during endurance (e.g., cross-country skiing), strength/power (e.g., luge), teamplay (e.g., ice hockey) or skill (e.g., curling) sports, but no data are currently available in youth athletes. Although, it is likely that macronutrient requirements will differ for individuals across growth and maturation as well as across different sports macronutrient recommendations are difficult to accurately prescribe without knowing the total daily energy requirements (Bergeron et al., 2015). Indeed, the main focus for practitioners working with youth winter sports athletes is to ensure the energy requirements for growth and maturation are met (Hannon et al., 2020b).

### Energy

A youth winter sports athlete's energy intake is provided through the consumption of the macronutrients, carbohydrate, fat, and protein (see Hannon et al., 2020b and, Desbrow et al., 2014 for further information and a more detailed review on macronutrient requirements for youth athletes in general). The energy intake of each athlete is dictated by their total energy expenditure (TEE) which comprises of three components: (1) basal metabolism (the energy required to maintain homeostatic physiology at rest); (2) thermic effect of food (the energy costs of digestion, absorption, transport, metabolism and storage of energy from food and drink), and (3) energy expenditure from planned physical activity and non-exercise activity thermogenesis (Food Agriculture Organization of the United Nations, 2004). In growing youth athletes, a fourth factor should be added representing the energy stored in their increasing body mass (e.g., increased fat-free mass) even though this latter factor is small (<100 kcal) on a daily basis (Prentice et al., 1988, Torun, 2005). Progressive increases in fat free mass (FFM), the most metabolically active body compartment, lead to increases in basal energy expenditure. Recent data from academy footballers showed progressive increases in resting metabolic rate from under 12 years (mean ± SD; 1699 ± 195 kcal·day^−1^) to under 16 years (2042 ± 155 kcal·day^−1^) age-groups after which there was no further increase in resting metabolic rate (Hannon et al., 2020a). Comparable resting metabolic rate data are lacking in youth winter sports athletes at different stages of growth and maturation. Before giving specific macronutrient recommendations, it is first essential to understand the typical energy expenditures experienced by youth winter sport athletes.

**Figure 1:**
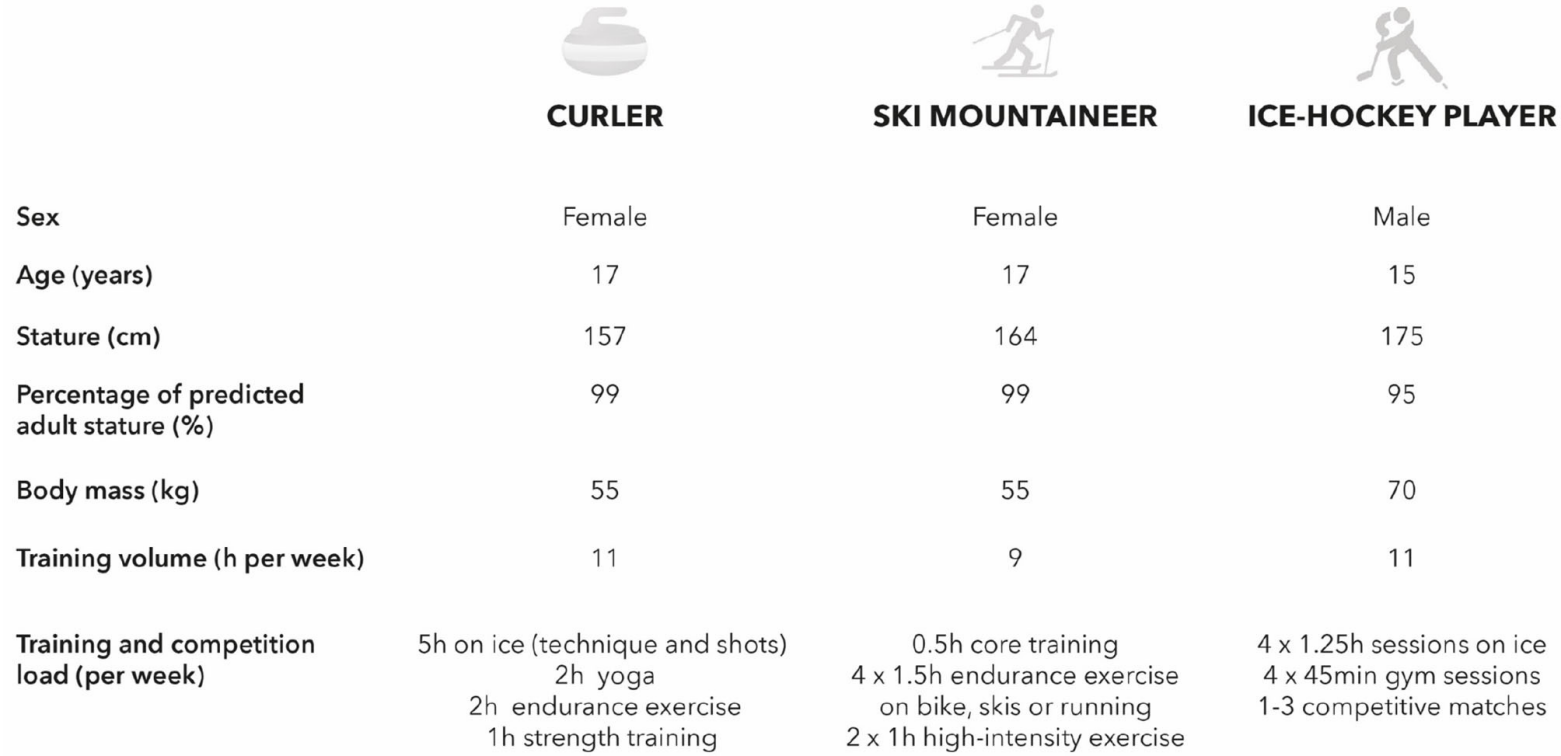
Sex, age, anthropometric characteristics and typical training schedule of a curler, ski mountaineer, and ice hockey player competing in the Lausanne 2020 Youth Olympic Games. The differences in anthropometric profile and training and competition loads between athletes likely result in differences in total energy expenditure and thus energy requirements. Data were kindly provided by the athletes and their coaches via a personal communication to V. Gremeaux.

In healthy physically active humans, exercise energy expenditure is the most variable contributor to TEE (Westerterp, 2013), and typically represents between 20 and 60% of TEE (Burke and Deakin, 2000). In athletes, especially endurance athletes, physical activity energy expenditure may become the greatest contributor to TEE (Torun, 2005, Silva et al., 2013). Exercise type, duration and intensity as well as an athlete's anthropometric profile will all influence exercise energy expenditure (and thus TEE). Indeed, there are large differences in the energy cost between different Olympic winter sports (Di Prampero et al., 1976, Tosi et al., 2010, Butte et al., 2018) due to differences in the physiological and metabolic demands of each specific sport (e.g., curling, figure skating, cross country skiing, ice hockey, ski mountaineering). Additionally, the different training loads of each sport change throughout adolescence (Balyi and Hamilton, 2004): for an example see the athlete career pathway by Swiss Olympic (https://www.swissolympic.ch/fr/federations/ftem-sport-athletenentwicklung.html). An overview of the typical training of three Swiss athletes at YOG in Lausanne 2020 is shown in Figure 1. Differences in their anthropometric profiles, and training and competition loads between these three athletes likely result in different total energy expenditures and thus energy requirements. Furthermore, training and competition loads may also vary across the annual training and competition cycle and between youth athletes of different ages competing in the same sport, which can lead to differences in total energy expenditure (Hannon et al., 2020c) and thus energy requirements. Across winter sports this results in a large inter-sport and inter-individual variability in total energy expenditure.

As an example, Ekelund et al. (2002) investigated the energy expenditure of youth speed-skaters (age range = 16–21 years) in pre-season and reported mean (±SD) daily total energy expenditure of 4037 ± 693 kcal·day^−1^ (~53 kcal·kg^−1^·day^−1^). Between athletes in this study (Ekelund et al., 2002) there was a difference of almost 3000 kcal in total daily energy expenditure, highlighting the need for an individual approach to energy requirements. No published data exist across different stages of growth and maturation in youth winter sports athletes using the gold standard doubly labeled water technique (Westerterp, 2017). Given the lack of direct measures of energy expenditure across different stages of maturation, precise nutritional guidelines (including macronutrient guidelines) for youth winter sports athletes is therefore difficult to formulate.

Whilst it is difficult to prescribe energy requirements for youth winter sports athletes at both the population and individual levels, it is advised that youth athletes have sufficient energy availability for growth. Energy availability is the amount of energy left for homeostatic physiological functions and growth once physical activity energy expenditure has been deducted from energy intake and is relative to FFM [energy availability = (energy intake–physical activity energy expenditure)/FFM]. Chronic low-energy availability (defined as <30 kcal·kg FFM^−1^·day^−1^ for adults) may lead to relative energy deficit in sport (RED-S), resulting in impaired growth and maturation of tissues and organs, reduced skeletal bone mineral accrual, increased risk of stress fractures, increased risk of osteoporosis later in life, delayed sexual maturation, disruption of reproductive function (i.e., menstrual dysfunction, low testosterone levels) and a less effective immune system (Loucks et al., 2011). Furthermore, low-energy availability can increase the risk of overreaching (Bellinger, 2020) and is associated with iron deficiency which may exacerbate some of the outcomes of low-energy availability such as sensation of fatigue (Sim et al., 2019). Not only is low-energy availability likely to have a negative effect on a youth athlete's sporting performance and development (Mountjoy et al., 2014, 2018a,b) it may also affect their long-term health. An energy availability of ≥45 kcal·kg FFM^−1^·day^−1^ is recommended for adult athletes to maintain normal physiological function (Loucks et al., 2011). Considering youth athletes have greater relative energy demands than adults, ≥45 kcal·kg FFM^−1^·day^−1^ is likely to be the minimum a youth athlete would require. Further research into energy availability especially in at-risk winter sport athletes (e.g., ski-jumpers) is required.

Winter sport training and competition often take place in cold environments and at moderate-to-high altitudes which have many physiological effects relevant to energy needs and intake (Meyer et al., 2011). During the Lausanne 2020 Youth Winter Olympic Games ambient temperatures and venue altitudes ranged from −10 to 14°C and from 400 to 2800 m, respectively. The primary mechanisms for altered energy needs and intake in the cold and at altitude include increases in energy expenditure and appetite suppression (Butterfield et al., 1992, Castellani et al., 2003, Matu et al., 2018). However, the relevance of these to the youth winter sports athlete remains unclear. For example, cold exposure time varies between events and is often counteracted by protective clothing and metabolic heat production during exercise (Bergeron et al., 2012). Furthermore, moderate altitude has been shown to increase resting metabolic rate in some (Woods et al., 2017b) but not all studies (Woods et al., 2017a). These points again highlight the potential value of obtaining accurate energy expenditure data in youth winter sports athletes at different stages of growth and maturation and across a variety of sports in order to formulate research-informed population specific nutritional guidelines. Nonetheless, it is difficult to prescribe energy intake on an individual level given there are many factors that influence a youth athlete's energy expenditure (and thus their energy requirements) including rate of growth, anthropometric profile and training and competition load. Indeed, there is a real risk of being too prescriptive regarding energy intake with youth athletes.

Total energy expenditure is a better starting point to prescribe dietary intake rather than diet macronutrient composition which is the basis of many sport nutrition recommendations (e.g., Desbrow et al., 2014). For example, in the study by Ekelund et al. (2002) the mean total energy expenditure of the youth ice-skaters (mean body mass = 75 kg) performing 1 h of training per day was 4000 kcal.day^−1^. Using the expert macronutrient recommendations by Desbrow et al. (2014) for adolescent athletes performing 1 h of training per day (3–5 g·kg^−1^·day^−1^ carbohydrate, 60 g carbohydrate per hour of exercise, 1.2–1.6 g·kg^−1^·day^−1^ protein and 20–35% energy intake from fat) the recommended energy intake value (3000 kcal) would underestimate the need for intake to cover actual energy expenditure and might lead to insufficient intake.

In summary, eating a varied and balanced diet that ensures sufficient energy availability for optimal growth and maturation as well as sporting performance is the cornerstone of youth athlete nutrition (Table 1). In addition, this should also allow for youth athletes to meet most of their micronutrient requirements, although some may require particular attention within this population.

## Micronutrients

Micronutrients are compounds that are required to maintain normal physiological function. They include minerals, vitamins, and several trace elements such as selenium. Although micronutrients do not directly supply energy for growth, maturation, and performance, they play essential roles in many metabolic pathways. There is currently no evidence to suggest that youth athletes have additional micronutrient requirements compared to their non-athletic peers. Four important principles should be taken into account. First, during growth and maturation there is increased need for some micronutrients; Second, micronutrient need does not linearly scale with increased physical activity energy expenditure; Third, increased physical activity expenditure is accompanied by increased nutritional intake and therefore also micronutrient intake (Heydenreich et al., 2017); And fourth, supplementation with micronutrients should be considered only when direct or indirect information points to a deficiency. Whilst it is essential that youth athletes consume adequate amounts of all micronutrients, there are certain vitamins and minerals that are of paramount importance such as calcium, vitamin D and iron.

### Calcium

Calcium is a crucial micronutrient for youth athletes, given that ~26% of bone mineral content accrues during peak bone mineral content accrual velocity (~12.5 and ~14 years old in girls and boys, respectively) and ~95% of adult bone mineral content is achieved by the end of adolescence (Bailey et al., 1999). Thus, calcium requirements of adolescents are increased as a result of the higher accrual of bone mineral content and during peak bone mineral content velocity, skeletal calcium accretion is ~300mg per day (Abrams et al., 2004). A number of studies however have reported low calcium intakes in both male and female youth athletes from a range of sports, which are significantly below the recommended daily amounts (e.g., Martinez et al., 2011).

Calcium requirements of adolescents have been described in detail elsewhere (Weaver et al., 2016) and to the best of our knowledge there are no considerations specific to youth winter sports athletes concerning calcium intake. In addition to calcium, the interplay with other nutrients such as phosphorus, magnesium and vitamin D is also vitally important for the formation of the mineral skeleton. Youth athletes are unlikely to be deficient in phosphorous and magnesium (Unnithan and Goulopoulou, 2004), but recent evidence suggests that youth winter sports athletes may be at increased risk of vitamin D deficiency (Zurcher et al., 2018).

### Vitamin D

Vitamin D (a prohormone) is a key regulator of calcium homeostasis, with sufficient levels required for calcium absorption (Holick, 2007). Therefore, sufficient vitamin D levels, along with calcium, is crucial to ensure maximal bone mineral accrual in youth winter sports athletes (Cashman et al., 2008). Vitamin D is primarily obtained through dermal synthesis upon exposure to ultraviolet-B (UVB) radiation. During winter months a lack of UVB radiation at high latitudes decreases vitamin D production in the skin (Holick, 2007). Vitamin D containing foods such as liver and eggs contain suboptimal quantities. Indeed, performing training in winter months and indoors (typical of youth winter sports athletes) increases a youth athlete's risk of vitamin D deficiency [serum 25(OH)D <30 nmol·L^−1^; Zurcher et al., 2018].

Vitamin D status (insufficient, deficient, sufficient or toxic; Ross et al., 2011) can be assessed using a simple blood test. If an athlete has serum vitamin D concentrations that are insufficient or deficient this can be corrected using supplemental vitamin D_3_. These decisions should be taken by a medical doctor and individualized to the athlete. Although a blanket approach to vitamin D_3_ supplementation is not advised, a common approach is to supplement athletes with during winter months even without testing vitamin D status (Owens et al., 2018).

### Iron

Iron is a trace element involved in many biological processes such as oxygen transport and energy metabolism (Beard, 2001, Hinton, 2014), and also plays a crucial role in the cognitive development of adolescents (Sachdev et al., 2005, Rouault, 2013). The main source is represented by dietary iron, which is poorly absorbed (Beard and Tobin, 2000). During childhood and adolescence, iron requirements increase as a result of tissue growth (Unnithan and Goulopoulou, 2004). Menarche in young women results in increased iron loss through menstruation, increasing iron requirements further in this population (Sandstrom et al., 2012). Exercise can result in iron loss through hemolysis, as well as in urine, stool, and sweat (Peeling et al., 2008). Iron deficiency is highly prevalent amongst adolescent athletes (up to 50% in females; Sandstrom et al., 2012), with inadequate dietary iron intake (often concomitant with inadequate energy intake or a vegetarian diet) often the main cause of iron deficiency (Mattiello et al., 2020).

Symptoms such as fatigue sensation and decreased performance can be associated to iron deficiency with or without anemia (Peeling et al., 2007). Improving iron status in deficient individuals can improve exercise efficiency (Hinton and Sinclair, 2007, Dellavalle and Haas, 2014), fatigue (Pratt and Khan, 2016) and recent evidence suggests that extra iron intake may be necessary for optimal erythropoietic adaptation to altitude (Garvican-Lewis et al., 2018, Hall et al., 2019). Thus, testing of youth athletes who present symptoms associated with iron deficiency (e.g., during regular medical check-up) or who are training at altitude will inform appropriate treatment strategies.

## Dietary Supplements

As discussed, supplementation may be necessary to correct for a clinically defined deficiency (e.g., vitamin D or iron deficiencies) and deficiency is generally the only appropriate reason for supplement use in youth athletes. A varied diet that includes sufficient intake of all the essential nutrients should always be the focus. All essential nutrients can be obtained in sufficient quantities from the diet alone with the exception of vitamin D (see section Vitamin D) albeit others may be challenging to obtain in sufficient quantities in certain diets (e.g., iron and vitamin B_12_ in a vegetarian diet). Nonetheless, the use of dietary supplements such as whey protein, carnitine, branched-chain amino acids, etc. is high amongst youth athletes (Evans et al., 2012). Whilst some of these supplements may provide a performance enhancing effect to some athletes, they come with a risk to health and of an anti-doping rule violation because of the undeclared presence of forbidden compounds in some (Maughan et al., 2004, Maughan et al., 2018). Rather than an attitude focusing on short-term results “potentially” aided by a supplement, youth athletes, their coaches, and parents should have a long-term commitment to good food optimisation which alongside training will provide the foundation for their athletic achievement.

## Hydration

Thermoregulation is a homeostatic process which regulates body temperature. Growth and maturation leads to decreases in an individual's surface-to-mass ratio and cutaneous blood flow, and increases sweating capacity (Falk and Dotan, 2011, Leites et al., 2016). Meaning, less mature adolescents rely more on radiative and conductive cooling rather than evaporative cooling (peripheral blood redistribution over sweating) to maintain thermal equilibrium (Falk and Dotan, 2011, Desbrow et al., 2014). Given the large inter-individual variability in fluid losses across adolescence, and that dehydration is a common challenge when working with young athletes (Arnaoutis et al., 2015) youth athletes should regularly monitor their fluid needs and consume as appropriate (see Table 1).

During exercise, thermoregulation prevents dangerous increases in core temperature. Exercising in hot and humid conditions (e.g., playing indoor ice-hockey) may be a risk for youth athletes as their core temperature seems to rise faster than adults under exercise or environmentally induced heat stress (Falk and Dotan, 2011). When training and competing in extreme conditions (i.e., either hot, humid or cold), youth athletes should take regular breaks and regularly consume cold and flavored fluids in hot conditions (Wilk and Bar-Or, 1996). Training in high altitude can further increase fluid loss at rest and during exercise through hypoxia-induced diuresis and hyperventilation increasing significantly water requirements at altitude to prevent dehydration (Butterfield et al., 1992). In addition, extremely high sweat rates and sodium losses have been reported for some sports such as ice hockey (~1.8 L·h^−1^) even though the training session was conducted in a cool environment (Palmer and Spriet, 2008, Gamble et al., 2019) highlighting again the effects of clothing on thermoregulation. Ice-hockey players are also at risk of dehydration due to repeated high-intensity efforts and limited fluid availability (Nuccio et al., 2017). It has been reported that junior ice hockey players did not drink enough fluid to prevent body mass losses of <2% during a game (Logan-Sprenger et al., 2011). Due to the large differences in sweat rates (and probably sweat composition) amongst youth athletes competing in different sports and in different environmental conditions, a blanket approach for hydration is unwise and individualized hydration strategies are advised (Table 1). It is recommended to monitor hydration status, to provide sufficient drinking opportunities to avoid dehydration and to rehydrate after exercise (e.g., at a rate of 1.5 L per kg body mass lost American College Of Sports et al., 2007).

## Education

Any discussion of nutrition for youth winter sports athletes would be incomplete without mentioning education. Indeed, holistic education is central to practitioners working with youth athletes in their quest to “develop healthy, capable, and resilient young athletes” (Bergeron et al., 2015). Nutrition education should not only focus on increasing nutrition knowledge but also on improving nutrition related skills e.g., budgeting, shopping, food hygiene, preparation, and cooking. This can be delivered in a variety of different formats including on an individual one-to-one basis, group workshops or remotely via photographs, infographics, videos etc. (Table 1). Nutrition education should also be extended to parents/guardians of youth athletes given that they are likely to be involved in food selection/preparation etc. as well as in reinforcing educational messages. Being overly prescriptive is by all means to be avoided. Given the extra-ordinary well-orchestrated and generally precisely tuned spontaneous regulation of energy balance, given a reasonably mixed healthy diet, one should part from having faith in physiology and let sensation inform the athlete, all the while accompanying, observing and evaluating, and only if objectively necessary guide and correct in an evidence-based manner.

## Conclusion

Youth winter sport athletes undergo rapid biological growth and maturation which influence their nutritional requirements. A sufficient energy intake is vital for optimal growth and development but also for sporting development. A varied and balanced diet (that ensures sufficient energy availability) should also allow for youth athletes to meet their micronutrient requirements. In some cases, vitamin D and iron should be monitored and supplemented (if appropriate) under the guidance of a medical professional. In addition to these specific nutritional needs, youth athletes, their parents, and coaches should be educated in sports nutrition. Adolescence is the “prime time” for qualified practitioners to provide athletes, parents, and coaches with information on research-informed nutritional practice. In any case, nutritional considerations for youth athletes should always put growth, maturation and sporting development first and performance second. Further research in youth winter sports athletes across different stages of growth and maturation competing in a variety of sports is now required in order to formulate research-informed nutritional guidelines for this population in keeping with this principle. Key research topics to be addressed include the energy, macro- and micronutrient requirements for youth athletes competing in different winter sports in addition to developing evidence informed educational strategies.

## Author Contributions

MPH and CD were invited to contribute to this special edition. MPH, JLF, VG and CD planned and drafted this manuscript. NP and BK provided critical revision of the manuscript. All authors approved the final version of this manuscript.

## Conflict of Interest

The authors declare that the research was conducted in the absence of any commercial or financial relationships that could be construed as a potential conflict of interest.
